# Preoperative Visual Attention Performance Predicts Postoperative Delirium

**DOI:** 10.1002/alz.091620

**Published:** 2025-01-03

**Authors:** Benjamin A Chapin, Faith Kimmett, Cynthia Garvan, Patrick Tighe, Steven Dekosky, John B. Williamson, Catherine C. Price

**Affiliations:** ^1^ Malcom Randall VA, Gainesville, FL USA; ^2^ University of Florida, Gainesville, FL USA; ^3^ UF, Gainesville, FL USA

## Abstract

**Background:**

Preexisting cognitive impairment is a significant risk factor for post operative delirium (POD), and POD increases morbidity and mortality. Disturbances of attention (i.e., ability to direct, focus, sustain, and shift attention) and awareness (i.e., orientation to environment) are necessary for delirium diagnosis. This suggests dysfunction in frontoparietal networks which control visuospatial attention. However, preoperative visual attention has not been systematically investigated as a risk factor for delirium in at‐risk adults.

**Methods:**

In this prospective observational study, participants aged 65 years and older undergoing elective orthopedic surgery completed preoperative measures of visuospatial attention, including horizontal line bisections and a preparation of the Posner cueing task in vertical and horizontal orientations. Our primary outcome was maximum POD severity, as measured by the Confusion Assessment Method Severity (CAM‐S) short form. Data were summarized (i.e., frequencies, mean (SD), and median [Q1, Q3]) and analyzed (i.e., Spearman correlational testing).

**Results:**

The majority of the 28 participants were female (68%) and white (93%) with mean (SD) age of 75.0 (6.1) years of age and 15.6 (2.7) years of education. 75% were not delirious post‐surgery, 11% were classified as subsyndromal (CAM‐S > 0), and 14% were classified as having POD. The median [Q1, Q3] value for the delirium score was 0 [0, 1.5]. The median [Q1, Q3] values for cognitive measures were as follows: Montreal Cognitive Assessment (MoCA) was 24 [22,27], line bisection leftward deviation was 0.12 mm [‐.40,0.11], incongruent stimulus reaction time was 550 ms [488,616]. Correlations between delirium and age, education, and MoCA score were not found to be significant. The correlation coefficient (p‐value) between delirium and leftward deviation on line bisection was 0.39 (p = 0.038), and incongruent stimulus reaction time was 0.57 (p = 0.003).

**Conclusion:**

We found that preoperative measures of visual attention predicted postoperative delirium. Further studies can help to determine how to apply these measures to better predict and monitor the effects of delirium on cognition and neurodegeneration. Understanding this relationship also provides a basis for future research on strategies to prevent, mitigate or rehabilitate the effects of delirium, improving hospital outcomes and potentially delaying or preventing dementia. Funding AACSF‐22‐928731; K07AG066813

